# Ocrelizumab-Induced Leukocyte Subpopulations Dynamics in Multiple Sclerosis

**DOI:** 10.3390/ph19101514

**Published:** 2026-09-23

**Authors:** Roberto De Masi, Stefania Orlando

**Affiliations:** 1 Laboratory of Neuroproteomics, Multiple Sclerosis Centre, “F. Ferrari” Hospital, 73042 Casarano, LE, Italy; 2 Complex Operative Unit of Neurology, “F. Ferrari” Hospital, 73042 Casarano, LE, Italy

**Keywords:** multiple sclerosis, leukocyte subpopulations, ocrelizumab, immunomodulation

## Abstract

**Background/Objectives**: Multiple sclerosis (MS) is a chronic autoimmune disease of the CNS characterized by immune-mediated inflammation and neurodegeneration. Based on its immunoablative effect targeting the CD20-positive B lymphocytes, ocrelizumab has emerged as a valid therapeutic option for MS. Although the on-treatment dynamics of leukocyte subpopulations are crucial for optimizing therapeutic strategies, little data are available in this field. Here, we aim to investigate how pre- and post-infusion immune-cell subsets change and correlate with each other, and how CD19+ B cell repopulation evolves over time. **Methods**: In an observational, retrospective, and prospective real-world study, we studied 51 relapsing-remitting MS ocrelizumab-treated patients who underwent flow cytometric immunophenotyping before and after each infusion and at three-month intervals, up to the eighth (4 years), for a total of 408 cycles. **Results**: After an initial decrease, post-infusion lymphocyte count returned to normal, while total white blood cells showed a sawtooth pattern driven by post-infusion increases. Granulocytes and natural killer cell counts remained substantially stable, while monocytes showed a significant cyclic pattern: increasing post-infusion, decreasing at the intermediate time point, with a more pronounced reduction pre-infusion, and rising again after re-administration. CD3+ and CD4+ T cells exhibited the same cyclic sawtooth pattern observed for monocytes. The CD4+/CD8+ ratio progressively increased, reflecting a significant elevation of CD4+, CD3+ and, to a lesser extent, CD8+ T cells. Pre-infusion CD19+ B cell counts progressively decreased, approaching zero by the fourth treatment year. Pre-treatment CD3+, CD4+, CD8+, lymphocyte, monocyte, and granulocyte counts differed significantly from on-treatment values. Correlation analyses showed that the major relationships among leukocyte subsets remained preserved throughout treatment. **Conclusions**: This study details leukocyte subpopulation dynamics during ocrelizumab therapy, unveiling previously unrecognized immunomodulatory properties beyond B-cell depletion. Ocrelizumab predominantly affects adaptive immunity, with potential indirect effects on innate immune compartments, and is associated with changes across immune-cell populations. These findings could have implications for individualized treatment strategies, but their direct application in this field still requires confirmatory studies on immunophenotyping and peripheral B-cell repopulation dynamics.

## 1. Introduction

Multiple sclerosis (MS) is a chronic autoimmune disease characterized by immune-mediated damage to the central nervous system (CNS), with both inflammatory and neurodegenerative components [1]. Although the exact etiology of MS remains elusive, accumulating evidence suggests that aberrant innate and adaptive immune responses, particularly those involving lymphocytes, play a crucial role in disease pathogenesis [2]. Recent advances in immunotherapy have revolutionized MS treatment, and among the emerging therapeutic options, ocrelizumab (Ocrevus, Genentech, Inc.—Roche Group, South San Francisco, CA, USA) (OCR), a humanized monoclonal antibody targeting CD20-positive B lymphocytes, has demonstrated significant efficacy in reducing disease activity and slowing disability progression. Following the OPERA I, OPERA II, and ORATORIO clinical trials, OCR was approved as a second-line disease-modifying treatment (DMT) for both relapsing-remitting MS (RRMS) and primary progressive MS (PPMS) [3,4].

This molecule selectively depletes CD20-positive B cells from the peripheral blood, modulating the immune response and potentially ameliorating the disease process. B cells contribute to MS pathology through various mechanisms, including antigen presentation, pro-inflammatory cytokine secretion, and pathogenic antibody production. By targeting these cells, OCR aims to interrupt the immune cascade underlying MS and consequently provide clinical benefits to patients [5,6].

Understanding the dynamics of lymphocyte subpopulations following OCR treatment is crucial for unraveling the drug’s mechanisms of action and optimizing therapeutic strategies for MS management. Flow cytometry, a powerful tool for detailed immunophenotyping, enables the accurate characterization and quantification of distinct lymphocyte subsets, based on specific cell surface markers. Through this technique, researchers have investigated the kinetics of peripheral lymphocyte subpopulations in MS patients treated with ocrelizumab as well as in patients receiving other anti-CD20 therapies, such as rituximab.

Indeed, CD19+ B cell count has emerged as a crucial indicator of treatment response in patients undergoing anti-CD20 therapies, as CD19 is a surface marker expressed on most normal and pathogenic memory B cells. Accordingly, a post-infusion CD19+ B cell count ≥1% is generally considered indicative of early B cell repopulation and may guide the timing of DMT reinfusion. However, this is not the case for OCR, which is administered at fixed six-month intervals regardless of B cell count. In any case, although B cell counts are not routinely monitored, gaining insight into lymphocyte subpopulation dynamics in ocrelizumab-treated MS patients may enhance our understanding of the immunological changes associated with this therapeutic approach, particularly regarding the impact of this therapy on the various lymphocyte subsets, including B cells, T cells, Natural Killer (NK) cells, CD4+ T helper cells, CD8+ cytotoxic T cells, and the CD4+/CD8+ ratio. In fact, although OCR was originally developed to selectively target CD20-positive B cells, increasing evidence suggests that its immunological effects extend beyond the B cell compartment [7]. Several studies have reported changes in T cell subsets, particularly CD8+ T cells and NK cell populations during treatment [8,9,10,11]. Moreover, emerging data indicate that the modulation of specific lymphocyte subsets may be associated with treatment response and long-term clinical outcomes [8,12,13]. Nevertheless, the kinetics of peripheral immune cell populations following OCR therapy remain incompletely characterized, and data on non-lymphoid leukocyte populations, including monocytes and polymorphonuclear leukocytes (PMN), remain very limited.

This article aims to provide a more comprehensive characterization of leukocyte dynamics, with a particular focus on lymphocyte subsets, in MS patients undergoing OCR treatment. Furthermore, it explores these changes in the context of MS pathophysiology and treatment response. Such knowledge lays the groundwork for treatment monitoring and individualized therapeutic management; however, the potential relevance of peripheral B-cell repopulation dynamics to treatment intervals requires prospective validation in a larger population.

## 2. Results

### 2.1. Overall Effects of Ocrelizumab Treatment on Leukocyte Subpopulations

All enrolled MS patients completed the eight OCR infusions, resulting in 408 analyzed treatment cycles overall. Baseline hematological parameters were normal in all patients. The overall effect of OCR therapy was assessed by comparison of mean pre-treatment (pre-OCR) with mean on-treatment (post-OCR) values obtained from all available assessments performed after treatment initiation and up to the eighth OCR infusion cycle (Figure 1A). In addition, the short-term effects of OCR administration, mean pre-infusion (pre-INF) (15 days before) and post-infusion (post-INF) (15 days after) values were also compared (Figure 1B). The corresponding descriptive and inferential statistics are summarized in Table 1. OCR treatment was associated with significant changes in T cell subpopulations, including increased CD3+, CD4+, and CD8+ relative and absolute counts, as well as a higher CD4+/CD8+ ratio. Similarly, pre-INF versus post-INF comparisons further revealed significant short-term variations in total white blood cells (WBC), lymphocytes, monocytes, PMNs, and CD3+ and CD4+ T cell absolute counts, while NK cells remained substantially unchanged.

### 2.2. Longitudinal Leukocyte Populations Dynamics

Descriptive statistics for all investigated leukocyte subsets, expressed as both cell frequencies (%) and absolute cell counts (c/µL), are provided in Supplementary Data S1. WBC counts displayed cyclical oscillatory changes throughout OCR treatment, characterized by recurrent post-infusion increases followed by declines before the subsequent infusion (Supplementary Figure S1). This fluctuating pattern became more evident in later infusion cycles, particularly after the sixth treatment cycle.

Total lymphocyte frequencies and absolute cell counts showed only minor fluctuations across treatment cycles (Supplementary Figure S2). However, even though isolated pairwise differences reached statistical significance, no clear temporal trend was observed.

Monocyte frequencies and absolute cell counts showed significant longitudinal variations during OCR treatment (overall longitudinal *p* = 0.0021 and *p* = 0.0028, respectively; Figure 2). Both parameters displayed a characteristic sawtooth pattern, with repeated post-infusion peaks followed by progressive reductions during the inter-infusion interval, further decreases at pre-infusion evaluations, and subsequent increases after each OCR administration. Several significant pairwise comparisons involved the post-2nd dose of the 1st infusion assessments.

PMN frequencies and absolute counts also showed cyclical fluctuations throughout OCR treatment, with alternating post-infusion increases and inter- and pre-infusion declines, while remaining overall stable across the study period (Supplementary Figure S3).

### 2.3. T-Cell Compartment Dynamics

CD3+ T cell frequencies increased rapidly after OCR initiation and remained persistently elevated throughout follow-up compared with baseline values (overall longitudinal *p* < 0.0001; Figure 3). Recurrent cyclical fluctuations were also evident across treatment cycles, characterized by post-infusion increases followed by inter-infusion declines. Absolute CD3+ T cell counts displayed a similar, although less pronounced, oscillatory pattern (Figure 3).

Consistent with the findings observed for total CD3+ T cells, CD4+ T cell frequencies increased significantly after OCR initiation and remained elevated throughout follow-up compared with baseline values (overall longitudinal *p* < 0.0001; Figure 4). Absolute CD4+ T cell counts also showed significant longitudinal variation over the study period (overall longitudinal *p* = 0.0483). This parameter exhibited in both cases a characteristic sawtooth pattern, with recurrent post-infusion increases followed by progressive inter-infusion declines and subsequent rises after each OCR administration.

CD8+ T cell frequencies remained relatively stable throughout follow-up, showing only limited variations across treatment cycles (Figure 5). Nevertheless, values were lower, particularly after the 6th infusion, coinciding with peak CD4+ T cell frequencies. Consistent with this divergent behavior of the two T cell subsets, the CD4+/CD8+ ratio increased during follow-up, reaching its highest values during the final study assessments (Figure 6).

NK cells remained substantially stable over time, with only limited variations across treatment cycles (Supplementary Figure S4), and no consistent longitudinal changes were observed.

### 2.4. CD19+ B Cells

In the longitudinal evaluation of the B cell subset, we observed a progressive decline in both pre-infusion CD19+ B cell frequencies and absolute cell counts throughout the observational period (*p* = 0.0371 and *p* = 0.0497, respectively; Figure 7). Notably, pre-infusion values continued to decrease over time, reaching near-zero levels by the fourth year of treatment, suggesting a cumulative depletion of circulating CD19+ B cells during OCR therapy.

### 2.5. Baseline and On-Treatment Correlations

We also examined correlation analyses across all investigated leukocyte subsets at baseline and during OCR treatment. Correlation matrices of these findings are shown in Supplementary Figure S5. Only selected biologically relevant correlations are presented in the main text, whereas the complete correlation matrices obtained using both cell frequencies (%) and absolute cell counts (c/µL) are provided in Supplementary Data S1. Percentage values were preferentially reported, as they describe the relative distribution of immune-cell subsets within the peripheral leukocyte compartment. Correlation patterns derived from absolute cell counts were largely consistent with those obtained with cell frequencies.

Spearman’s correlation analysis revealed significant associations among major leukocyte populations. Specifically, lymphocytes pre-OCR were inversely correlated with PMNs both at baseline (*r* = −0.8980, *p* < 0.0001) and after OCR initiation (*r* = −0.6856, *p* < 0.0001). Furthermore, lymphocytes post-OCR were negatively associated with post-OCR PMNs (*r* = −0.9749, *p* < 0.0001).

Baseline CD3+ T cells showed a positive correlation with both pre- and post-OCR CD8+ T cells (*r* = 0.4019, *p* = 0.0092; *r* = 0.3985, *p* = 0.0099) and inversely with both pre- and post-OCR NK cells (*r* = −0.6861, *p* < 0.0001; *r* = −0.6295, *p* < 0.0001, respectively). Similarly, post-OCR CD3+ T cells remained strongly correlated with both post-OCR CD8+ T cells (*r* = 0.5337, *p* = 0.0003) and NK cells (*r* = −0.9580, *p* < 0.0001). Baseline CD4+ T cells were inversely associated with both pre- and post-treatment CD8+ T cells (*r* = −0.4794, *p* = 0.0015; *r* = −0.4331, *p* = 0.0047) and both pre- and post-OCR CD4+/CD8+ ratios (*r* = 0.7222, *p* < 0.0001; *r* = 0.5796, *p* < 0.0001, respectively). These correlations remained strongly significant when post-OCR CD4+ T cells were considered (*r* = −0.6292, *p* < 0.0001; *r* = 0.8008, *p* < 0.0001, respectively).

Baseline CD8+ T cells correlated inversely with post-OCR CD4+ T cells (*r* = −0.6490, *p* < 0.0001) and with pre- and post-OCR CD4+/CD8+ ratios (*r* = −0.9389, *p* < 0.0001; *r* = −0.8623, *p* < 0.0001, respectively). Likewise, post-treatment CD8+ T cells remained strongly and negatively associated with the post-treatment CD4+/CD8+ ratio (*r* = −0.9607, *p* < 0.0001).

Consistent with these findings, baseline CD4+/CD8+ ratio directly correlated with post-treatment CD4+ T cells (*r* = 0.7570, *p* < 0.0001) and inversely with post-treatment CD8+ T cells (*r* = −0.7909, *p* < 0.0001), further supporting that the longitudinal increase in the CD4+/CD8+ ratio was driven by the divergent behavior of the CD4+ (with a considerable increase) and CD8+ T (with a slight increase) cell compartments.

Baseline NK cells were also inversely correlated with post-OCR lymphocytes (*r* = −0.4545, *p* = 0.0028), post-OCR CD3+ (*r* = −0.7071, *p* < 0.0001) and CD8+ T cells (*r* = −0.4537, *p* = 0.0029) and directly with post-OCR CD4+/CD8+ ratio (*r* = 0.3281, *p* = 0.0363).

### 2.6. Pre- and Post-Infusion Correlations

We next examined the correlations among immune-cell subsets by comparing pre-, inter-, and post-infusion assessments during the OCR administration period. Correlation matrices including these three time points are provided in Supplementary Figure S6. As in the previous analysis, only selected biologically relevant correlations between pre- and post-infusion assessments based on percentage values are presented in the main text, whereas the complete correlation matrices obtained using both cell frequencies (%) and absolute cell counts (c/µL) are provided in Supplementary Data S1.

Spearman’s rank analysis across individual OCR infusions confirmed the correlation patterns observed at OCR initiation, and also in the following study period, indicating that the relationships among the major leukocyte subsets were consistently maintained over time after the initial administration of the drug.

In particular, lymphocytes remained strongly and inversely associated with PMNs, regardless of the timing of assessment, including both pre- and post-INF evaluations (pre/pre: *r* = −0.9617; pre/post: *r* = −0.7854; post/post: *r* = −0.9208, respectively; all *p* < 0.0001).

Similarly, CD3+ T cells directly correlated with CD8+ T cells both before infusion (*r* = 0.5344, *p* = 0.0002), across pre-/post-infusion assessments (*r* = 0.5268, *p* = 0.0003), and after infusion (*r* = 0.5568, *p* = 0.0001). Conversely, CD3+ T cells showed a strong inverse correlation with NK cells throughout the infusion cycle (pre/pre: *r* = −0.9456; pre/post: *r* = −0.8586; post/post: *r* = −0.9672, respectively; all *p* < 0.0001).

Likewise, CD4+ maintained their inverse correlation with CD8+ T cells and direct correlation with the CD4+/CD8+ ratio, both before and after infusion (pre/pre: *r* = −0.5504 and *r* = 0.7195; pre/post: *r* = −0.5501 and *r* = 0.6855; post/post: *r* = −0.6257 and *r* = 0.7814; all *p* < 0.0001). Consistently, pre-INF CD8+ T cells were directly correlated with post-infusion CD3+ T cells (*r* = 0.5744, *p* < 0.0001) and inversely with both CD4+ T cells in pre- and post-INF assessment (*r* = −0.5504, *p* < 0.001; *r* = −0.6000, *p* < 0.0001, respectively) and CD4+/CD8+ ratio (*r* = −0.9585, *p* < 0.0001; *r* = −0.9293, *p* < 0.0001). Comparable association patterns were observed in the post-infusion analysis over time. Specifically, post-INF CD8+ T cells remained strongly and inversely correlated with the CD4+/CD8+ ratio (*r* = −0.9661, *p* < 0.0001). Likewise, the pre-INF CD4+/CD8+ ratio remained directly correlated with post-INF CD4+ T cells (*r* = 0.7350, *p* < 0.0001) and inversely with post-INF CD8+ T cells (*r* = −0.9448, *p* < 0.0001), confirming the persistence of the reciprocal, long-term CD4+/CD8+ relationship across infusion cycles.

Pre- and post-INF NK cells also maintained significant inverse correlations with post-INF CD3+ T cells (*r* = −0.9177, *p* < 0.0001; *r* = −0.9672, *p* < 0.0001, respectively) and CD8+ T cells (*r* = −0.4394, *p* = 0.0032; *r* = −0.4977, *p* < 0.0001, respectively). Taken together, these findings indicate that several associations among the major leukocyte subsets were consistent across the different assessment periods. In addition, these correlation patterns were observed both before and after OCR infusion.

Finally, selected biologically relevant correlations are further illustrated by representative linear regression analyses in Figure 8.

## 3. Discussion

OCR is a highly effective treatment in MS due to the selective depletion of CD20+ B cells [3,5,6]. However, its effectiveness is not limited to this mechanism of action. Previous studies have reported modest and heterogeneous effects of OCR on peripheral T cell subsets, particularly CD4+ and CD8+ T cells, highlighting the need for long-term longitudinal analyses [7,10].

This study provides a comprehensive longitudinal evaluation of peripheral blood immune-cell dynamics in patients with MS receiving a uniform OCR treatment regimen, followed for up to eight infusions (408 cycles overall). The homogeneous treatment protocol and the normal baseline hematological profile of all enrolled patients allowed for a clear interpretation of treatment-related immunological changes and leukocyte subpopulations dynamics. Furthermore, the large molecular size of this DMT provides a useful model for investigating how therapeutic modulation of peripheral immunity may relate to central neuroinflammation or peripheral complications, including colitis or psoriasis [14,15]. Despite the importance of these topics, no data are available that are consistent with an underlying evidence-based theory in the field. However, here we found significant correlations between the assessed variables, shedding light on the effects of OCR treatment on leukocyte subpopulations. In addition, we identified a novel DMT-induced pattern among leukocyte subsets throughout the observed treatment period. These findings provide further insight into the coordinated remodeling of peripheral immune-cell populations induced by OCR. These observations are consistent with our recent study on OCR-associated brain volume dynamics [16], suggesting that the biological effects of OCR extend beyond selective B cell depletion. Furthermore, we considered not only the patients with complete pre-infusion CD19+ B cell depletion, but also those showing measurable B cells during therapy, in order to better characterize repopulation kinetics during long-term OCR treatment.

Our data indicate that OCR induces a dynamic change in circulating immune cells, with reproducible patterns across treatment cycles. The most evident finding was the cyclical pattern observed over the treatment period, characterized by transient post-infusion changes followed by a gradual return toward pre-infusion values before the subsequent administration. This oscillatory behavior was particularly evident for total WBC, monocytes, PMNs, CD3+ and CD4+ T cells, whereas lymphocyte and NK cell counts remained comparatively stable over time.

Specifically, lymphocyte frequencies showed only a modest transient reduction immediately after treatment initiation, but absolute counts remained overall stable throughout follow-up, suggesting that the treatment does not cause persistent lymphopenia. Instead, it is associated with dynamic changes in lymphocyte subset proportions over time. In particular, a decrease in lymphocyte count of approximately 10% is observed starting after the first infusion. This percentage remains constant over time, in contrast to total WBC counts, which evidenced the cyclical oscillations typically associated with successive infusion cycles. This sawtooth pattern in total WBC counts, consistent with transient post-infusion elevation, further supports cyclical immune modulation tied to the timing of assessment rather than cumulative hematologic suppression. In fact, the recurrent post-infusion increases in WBCs mainly reflected a dynamic response within the innate immune compartment, with monocytes and PMNs showing reproducible post-treatment expansions followed, in turn, by progressive declines during the successive inter-infusion period.

Monocytes and PMNs showed a distinct kinetic profile compared with total lymphocyte counts. Both populations, in fact, showed early transient increases in post-treatment values, reaching the baseline values successively, although with intermittent peaks corresponding to specific infusion cycles. This pattern may reflect a systemic redistribution or mobilization phenomenon of the innate immune compartment, according to a conceptual analogy with plasmapheresis, where removal of circulating humoral components is followed by redistribution, although the underlying mechanisms involved are clearly distinct. However, the present data do not allow conclusions regarding functional activation.

Moreover, unlike NK cell frequencies, which remained substantially stable, monocyte frequencies increased by approximately 9%, remaining elevated throughout the treatment period. The evolution kinetics of monocytes and PMNs, as well as the temporal dissociation between their peaks, further suggests that these myeloid subsets may be regulated by independent signaling pathways, at least partly different from those of adaptive immunity.

As regards the adaptive immune compartment, OCR treatment was associated with a progressive increase in CD3+ and CD4+ T cell frequencies, sustained by an elevation of the CD4+/CD8+ ratio, whereas CD8+ T cells showed comparatively smaller changes. Notably, absolute cell counts showed cyclic infusion-related oscillations, while percentage values more consistently reflected uniform, long-term lymphocyte dynamics. This observation suggests the preferential use of relative frequencies for evaluating leukocyte changes during OCR treatment.

Within the lymphocyte compartment, a key finding is the progressive and stable increase in the CD4+/CD8+ ratio over time. This shift was driven primarily by a sustained increase in CD4+ and CD3+ T cells, with CD8+ T cells as a minor contributor. Importantly, this pattern is supported by a real, non-relative increase, since the absolute values also increased. In fact, while CD3+ and CD4+ T cell frequencies increased by approximately 16% and 19%, respectively, the CD4+/CD8+ ratio increased by approximately 7% during the follow-up period. Apart from these, no other changes were observed in T cell subsets when comparing the pre-treatment period with the on-treatment one, with the notable exception of CD19+ B cells.

Furthermore, pre-infusion CD19+ cell counts progressively declined over time, approaching zero by the fourth year of treatment, suggesting a sustained, cumulative B cell depletion effect of OCR. Although this progressive pre-infusion depletion raises questions regarding the timing of peripheral B cell repopulation and its potential relevance to individualized treatment, these implications require extensive prospective validation. Furthermore, the absence of functional immune-cell phenotyping, paired CSF and bone marrow indicators, and the lack of healthy and treatment-comparator groups limit the clinical interpretation of these findings. The inclusion of these compartments would have been invaluable, especially considering that the elements circulating in peripheral blood represent no more than 2% of the total pool. However, to fill this gap, while also addressing the related ethical constraints, a dedicated and independent study would be necessary.

While our cohort included only patients with RRMS, similar peripheral immune cell changes have also been reported in patients with PPMS treated with ocrelizumab, including B cell depletion and remodeling of the T cell compartment [11]. However, given the different clinical and immunopathological features of PPMS, the immune cell dynamics observed in our RRMS cohort cannot be directly extrapolated to progressive disease. Further longitudinal studies are needed to determine whether these patterns are shared across different MS phenotypes.

The immune cell dynamics observed during OCR treatment should also be considered in the context of other anti-CD20 therapies used in MS. Comparative studies have shown that rituximab and OCR induce a similar B cell depletion, while OCR may have a greater impact on CD4+ and CD8+ T cells [17]. Similarly, comparisons between OCR and ofatumumab have reported differences in B and T cell subset dynamics, although several lymphocyte populations, including CD4+ and NK cells, showed comparable changes [18,19]. Recent longitudinal data on ublituximab have also demonstrated transient post-infusion changes in CD3+, CD4+, CD8+ and NK cells, with persistent B cell depletion [20]. In particular, ublituximab is a glycoengineered anti-CD20 monoclonal antibody with enhanced affinity for FcγRIIIa (CD16), which may contribute to distinct effects on Fc-mediated effector cells [21]. These findings suggest that, despite a class effect due to the shared CD20 target, different anti-CD20 agents can induce partially distinct patterns of peripheral immune cell dynamics, depending on the degree of humanization of the active molecule and its ability to activate the CD20 target elimination process. The effectiveness of the drug comes from the first factor; from the second, the selectivity of action. In fact, unlike complement-dependent cytotoxicity (CDC), antibody-dependent cellular cytotoxicity (ADCC) is highly selective and specific. It is already known that OCR and ublituximab are engineered to preferentially mediate ADCC, while rituximab and ofatumumab preferentially mediate CDC, leading to different cytotoxic profiles [22].

Inferential statistical analyses revealed several consistent correlations among immune cell subsets. The use of percentage values revealed particularly informative in this context, describing well the relative distribution occurring among leukocyte populations. Most importantly, the major correlations among leukocyte subsets were consistently preserved both after OCR initiation and throughout successive infusion cycles. In fact, the inverse correlations between lymphocytes and PMNs were consistently observed across the different assessment periods.

In particular, the strong inverse correlations in both pre- and post-infusion regimens between CD3+ T cells and NK cells remained remarkably stable across all assessment settings, despite OCR-induced changes in each relative compartment. Also, the inverse correlation between CD3+ T cells and the CD4+/CD8+ ratio is consistent with both a mathematical and biological relationship between these variables. CD4+ and CD8+ T cells exhibited complementary and reciprocal correlation patterns with PMNs, NK cells, and the CD4+/CD8+ ratio.

Conversely, monocytes showed less consistent correlations with the other leukocyte subsets. Monocytes, in fact, are considered elements of the immune system, belonging half to innate and half to adaptive immunity. Importantly, correlations between their pre- and post-infusion regimens indicate a relationship between baseline and post-infusion profiles of the other leukocyte elements.

Linear regression analyses further supported these observations, confirming a statistical relationship between pre- and post-infusion leukocyte profiles. Consistently, pre-infusion levels of the major leukocytes were associated with their corresponding post-infusion values, as confirmed by linear regression analyses.

These findings are in line with our previous observations highlighting the value of predictive biomarkers for treatment response in MS [23,24,25,26].

Notably, the observed associations among leukocyte subpopulations describe relationships underlying the peripheral immune-cell profile, although circulating lymphocytes constitute less than 2% of the total pool [27]. Consequently, it is already known that the described systemic CD4+/CD8+ shift is pathogenetically linked to systemic lupus erythematosus, sarcoidosis, and HIV infection [28,29,30], but underlying CD8+ dysregulation may also be relevant for OCR-induced colitis or psoriasis [14,15].

Finally, our findings indicate that consistent individual patterns of peripheral immune-cell measurements were observed despite treatment-induced changes, which may contribute to the interindividual variability of immunological profiles.

## 4. Materials and Methods

### 4.1. Study Design

This observational, retrospective and prospective real-world study enrolled 51 consecutive OCR-treated RRMS patients attending the Multiple Sclerosis Centre—Department of Neurology at the “F. Ferrari” Hospital, Casarano, Lecce, Italy. Laboratory data were collected both retrospectively and prospectively; patients were followed longitudinally up to the eighth OCR treatment cycle (approximately four years of therapy). According to the drug schedule, each enrolled subject underwent the drug infusion every six months, consisting of 600 mg administered in 500 mL of 0.9% NaCl solution, except for the first cycle, which was given in two 300 mg infusions in 250 mL of 0.9% NaCl solution, two weeks apart. Patients were subjected to hematological analysis and flow cytometry on peripheral venous blood samples, collected 15 days before (pre-INF) and after (post-INF) each drug infusion, with an additional intermediate evaluation performed every three months. At each assessment, WBCs, monocytes, PMNs, and lymphocyte subpopulation counts were recorded. Flow cytometric analyses carried out quantitative assessment of CD3+ T lymphocytes, CD3+CD4+ T helper lymphocytes, CD3+CD8+ T cytotoxic lymphocytes, CD3-CD19+ B lymphocytes, CD3-CD56+ NK cells, and the CD4+/CD8+ ratio. Both relative proportions (% of WBCs or lymphocytes) and absolute cell counts (cells/μL) were collected.

### 4.2. Study Population

Subjects’ enrollment took place at the MS Centre of Casarano, Lecce (Italy), during routine visits, according to predefined inclusion and exclusion criteria.

Inclusion criteria: OCR-treated RRMS patients from the start of therapy and followed longitudinally thereafter up to the eighth drug infusion. All MS patients were previously diagnosed according to the 2017 McDonald criteria [31] and examined in an exacerbation-free period of at least three months. Written informed consent was obtained from all participants.

Exclusion criteria: Any metabolic, cardiovascular, or immunological comorbidity (atheromatosis or prior stroke, diabetes, arteritis, rheumatoid arthritis, or connective tissue disorders), local or systemic transient inflammatory and septic conditions (bacterial infections, cold, cough, influenza, exanthematous diseases, viral infections, relevant trauma, smoking, or obesity). Patients receiving any non-MS treatment within at least three months prior to enrollment and those expressing abnormal cell count at the hemocromocytometric examination before the study entry were also excluded. All these conditions are considered confounding factors, capable of altering the leukocyte count as a pro-inflammatory systemic bias.

### 4.3. Cytofluorimetric Assessment

Whole venous blood samples were analyzed using Navios EX Flow Cytometer (Beckman Coulter, Ireland Inc., Lismeehan O’Callaghan’s Mills Co. Clare, Ireland) and Navios EX software (version 2.2, Beckman Coulter). For flow cytometric analysis, 100 μL of whole blood was incubated for 10 min at room temperature with specific combinations of fluorochrome-conjugated monoclonal antibodies directed against the following cell-surface antigens: CD45-FITC (fluorescein isothiocyanate), CD4-RD1 (phycoerythrin), CD8-ECD (phycoerythrin-Texas Red-X), CD3-PC5 (phycoerythrin-Cy5), CD56-RD1 (phycoerythrin) and CD19-ECD (phycoerythrin-Texas Red-X), using 10 μL of two CYTO-STAT tetraCHROME panels for each tube (CD45-FITC/CD4-RD1/CD8-ECD/CD3-PC5 and CD45-FITC/CD56-RD1/CD19-ECD/CD3-PC5, both from Beckman Coulter).

Following red blood cell lysis with the COULTER IMMUNOPREP Reagent System on a TQ-Prep Workstation (Beckman Coulter), samples were analyzed by flow cytometry, using lymphocyte gates identified by their characteristic bright CD45 fluorescence and low side-scatter (SSC) properties. Leukocyte differential counts, including monocytes and granulocytes, were obtained from routine hematological analyses, while lymphocyte subsets were characterized by flow cytometry. The results of the two methodologies were combined to evaluate WBC subpopulation dynamics during ocrelizumab treatment.

### 4.4. Statistical Analyses

Statistical analysis was performed with GraphPad Prism 9 (GraphPad Software, San Diego, CA, USA). Descriptive statistics are presented as mean ± standard deviation (SD). The RM one-way ANOVA, with the Geisser-Greenhouse correction, was applied to analyze repeated measures, with a mixed-effects model used when missing values were present. Sidak’s correction was applied for multiple comparisons, where appropriate.

Spearman’s rank test was used to determine non-parametric correlations among immune-cell subsets and the correlation matrix for multiple variables analysis. Linear regression analysis was performed to assess the relationships between variables. Statistical significance was set at *p* < 0.05.

## 5. Conclusions

In conclusion, we demonstrate that OCR treatment induces dynamic changes in peripheral immune-cell populations over time, rather than selectively ablative or progressive immunosuppression. Beyond the known CD20+ B cell depletion, our findings suggest that OCR not only depletes memory B cells while sparing pre-B cells, as previously described in the literature, but is also associated with changes in other immune-cell populations, predominantly involving adaptive immunity. Specifically, the OCR treatment was associated with transient, infusion-related oscillations in immune cell counts, sustained depletion of CD19+ B cells, and a stable increase in the CD4+/CD8+ ratio, driven predominantly by CD4+ T cells. Several correlations between immune cell subsets were consistently observed in both pre- and post-infusion assessments over the study period. This means that the drug affects adaptive immunity, regardless of the assessment time-point, whether close to or distant from the administration period. Furthermore, the strong associations between baseline and post-OCR immune profiles suggest that pre-treatment immunophenotyping may provide useful information on successive individual immune responses to therapy.

These findings improve characterization of associations among leukocyte subsets during OCR treatment and highlight the potential value of longitudinal peripheral B cell monitoring. Future studies, integrating clinical and paraclinical outcomes with longitudinal immunophenotypic data, may help clarify the biological and prognostic implications of leukocyte dynamics and determine whether peripheral B cell repopulation dynamics may be useful in precision medicine. Although data on OCR discontinuation or modification of its dosing regimen are currently lacking, our results demonstrate CD19+ repopulation kinetics and immune dynamics related to DMT that may be relevant for future studies on personalized long-term therapeutic management in MS.

## Figures and Tables

**Figure 1:**
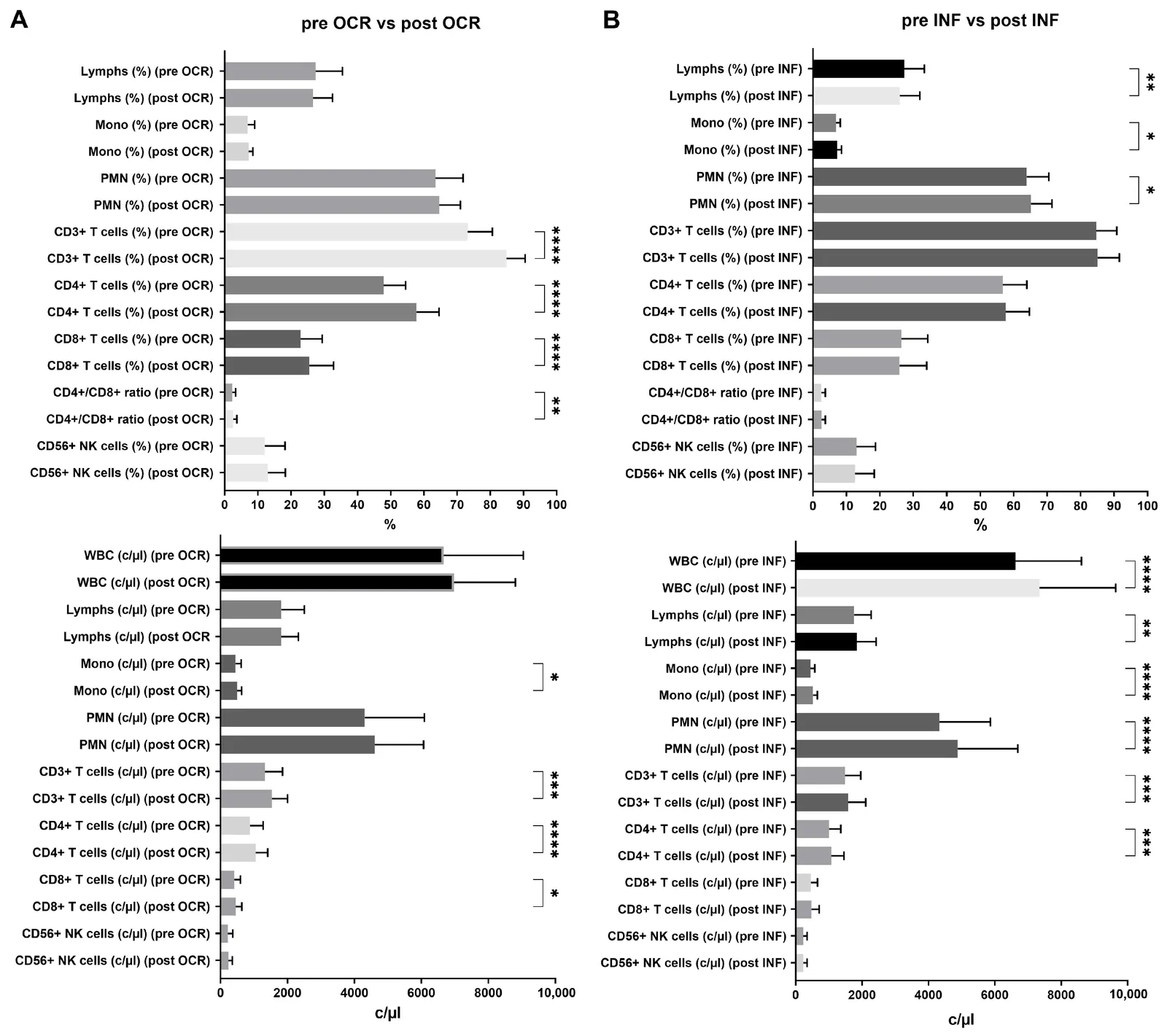
Overall effects of Ocrelizumab treatment and infusion-related changes on leukocyte subpopulations. (**A**) Comparison of mean pre-treatment (pre-OCR) and on-treatment (post-OCR) values and (**B**) pre-infusion (pre-INF, 15 days before infusion) and post-infusion (post-INF, 15 days after infusion) values across the entire cohort of 51 patients. Relative frequencies (% of WBCs) are shown in the upper panel, and absolute cell counts (cells/μL) in the lower panel. WBC—white blood cells, PMN—polymorphonuclear leukocytes. * = *p* < 0.05, ** = *p* < 0.01, *** = *p* < 0.001, **** = *p* < 0.0001.

**Figure 2:**
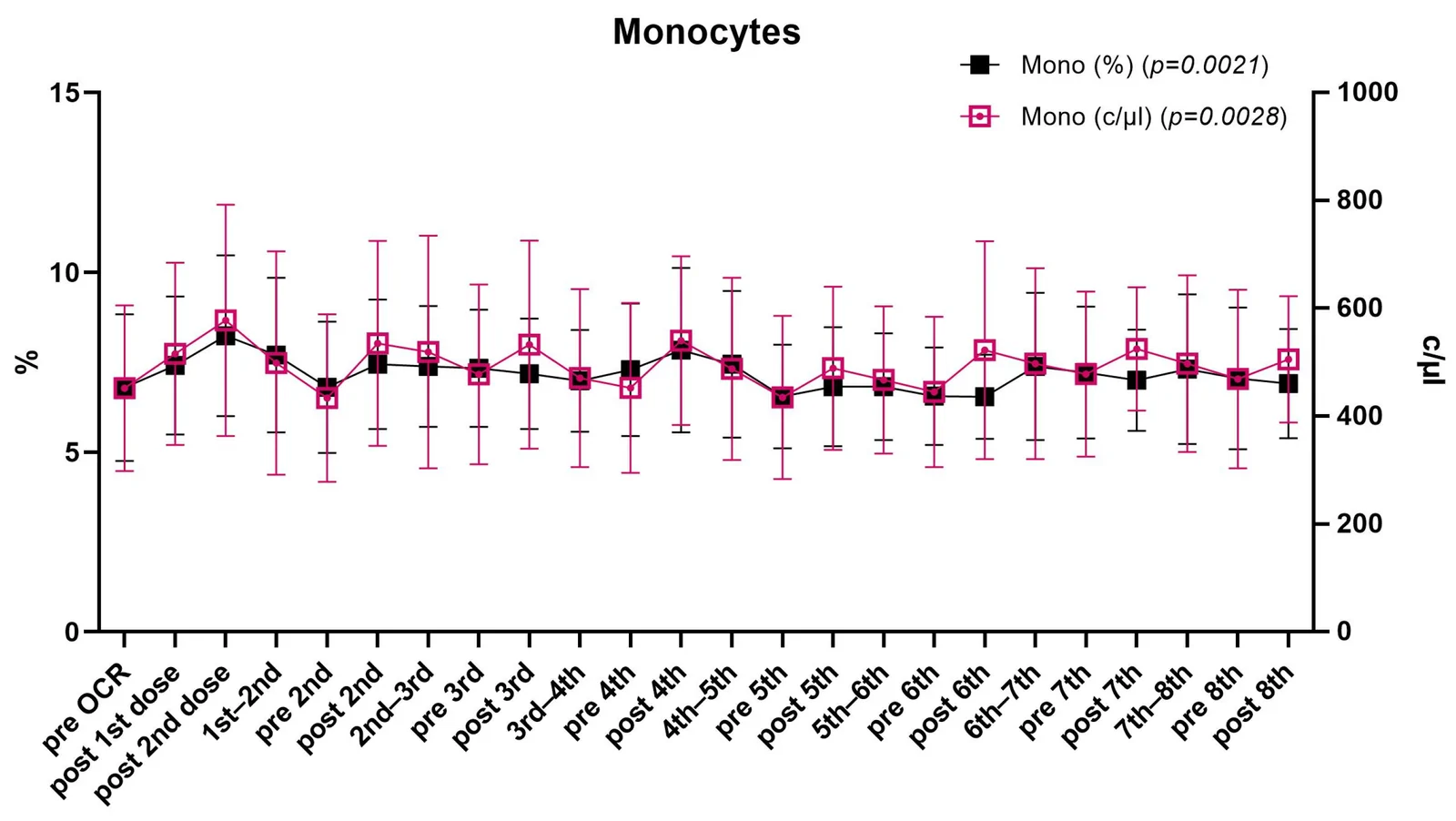
Longitudinal dynamics of monocytes during OCR treatment. Mean ± SD monocyte frequencies (% of total WBC) and absolute cell counts (c/µL) measured at each study time-point throughout eight OCR infusions. Note that both parameters showed a recurrent peak-and-decline profile across consecutive cycles. Overall longitudinal *p*-values were 0.0021 and 0.0028, respectively. Pre-OCR: baseline assessment before ocrelizumab start; post 1st dose and post 2nd dose: evaluations performed after the first and second doses of the initial infusion, respectively; time-points indicated by two consecutive infusion cycles (e.g., 2nd–3rd, 3rd–4th) represent intermediate inter-infusion assessments; thereafter, pre and post indicate evaluations performed 15 days before and 15 days after each subsequent OCR infusion, respectively.

**Figure 3:**
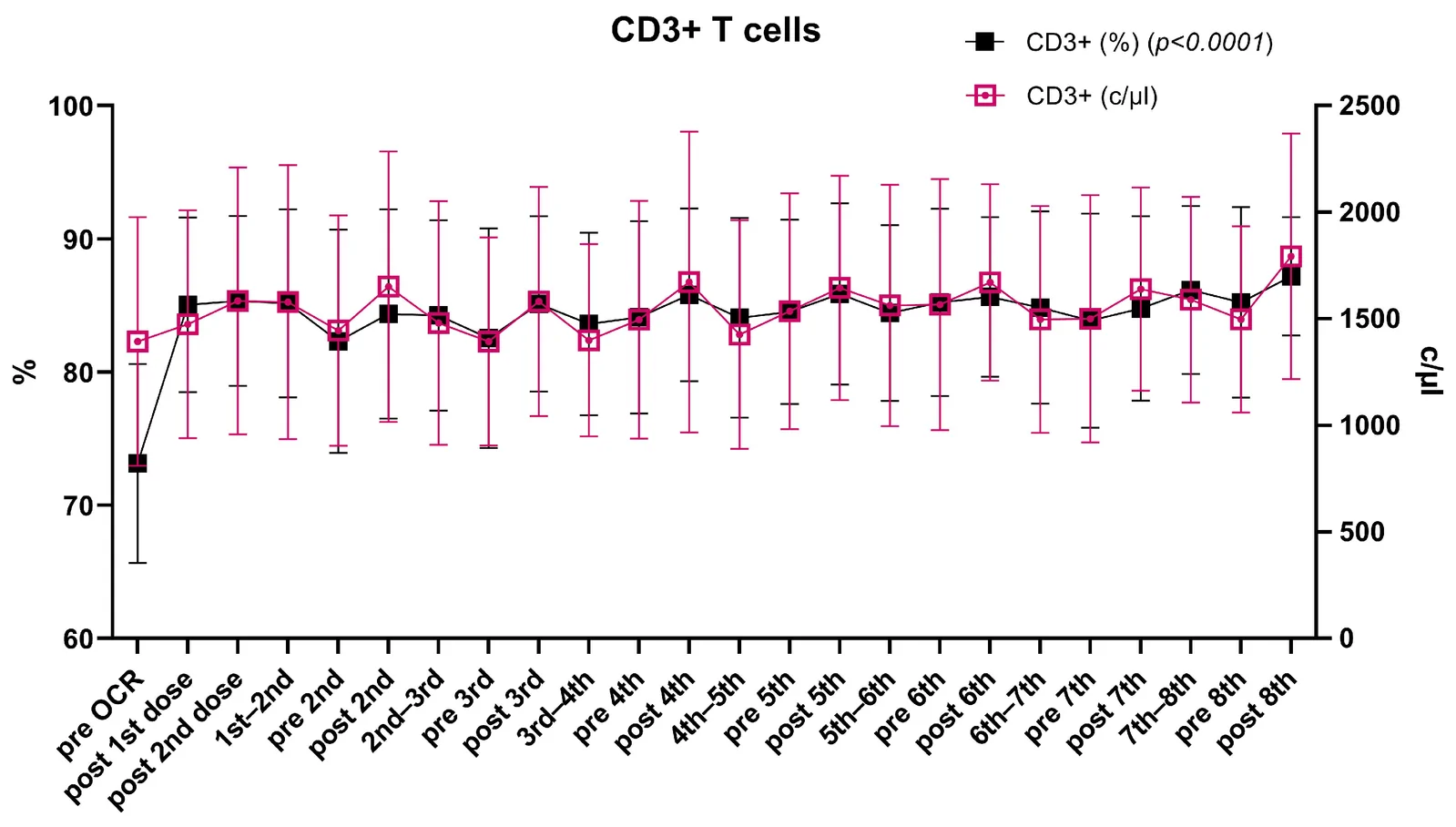
Longitudinal dynamics of CD3+ T cells during OCR treatment. Mean ± SD CD3+ T cell frequencies (% of total lymphocytes) and absolute cell counts (c/µL) measured at each study time-point throughout eight OCR infusions. Note that CD3+ T cell frequencies increased after OCR initiation and remained elevated throughout follow-up (overall longitudinal *p* < 0.0001), while both frequencies and absolute counts exhibited cyclical fluctuations across consecutive treatment cycles. Pre-OCR: baseline assessment before ocrelizumab start; post 1st dose and post 2nd dose: evaluations performed after the first and second doses of the initial infusion, respectively; time-points indicated by two consecutive infusion cycles (e.g., 2nd–3rd, 3rd–4th) represent intermediate inter-infusion assessments; thereafter, pre and post indicate evaluations performed 15 days before and 15 days after each subsequent OCR infusion, respectively.

**Figure 4:**
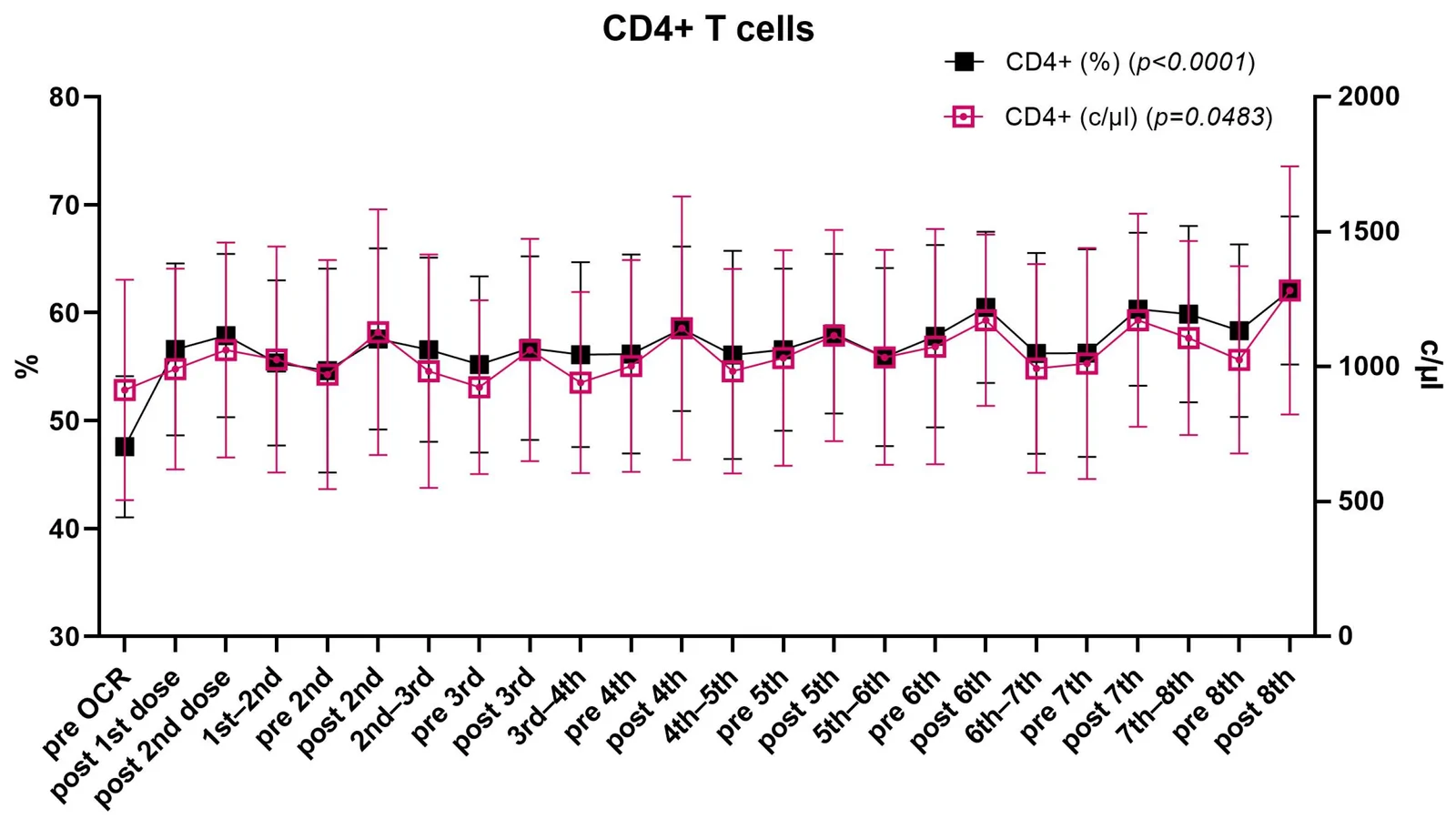
Longitudinal dynamics of CD4+ T cells during OCR treatment. Mean ± SD CD4+ T cell frequencies (% of total lymphocytes) and absolute cell counts (c/µL) measured at each study time-point throughout eight OCR infusions. Note that both parameters exhibited a characteristic sawtooth pattern, with recurrent post-infusion increases followed by progressive inter-infusion declines and subsequent rises after each OCR administration (overall longitudinal *p* < 0.0001 and *p* = 0.0483, respectively). Pre-OCR: baseline assessment before ocrelizumab start; post 1st dose and post 2nd dose: evaluations performed after the first and second doses of the initial infusion, respectively; time-points indicated by two consecutive infusion cycles (e.g., 2nd–3rd, 3rd–4th) represent intermediate inter-infusion assessments; thereafter, pre and post indicate evaluations performed 15 days before and 15 days after each subsequent OCR infusion, respectively.

**Figure 5:**
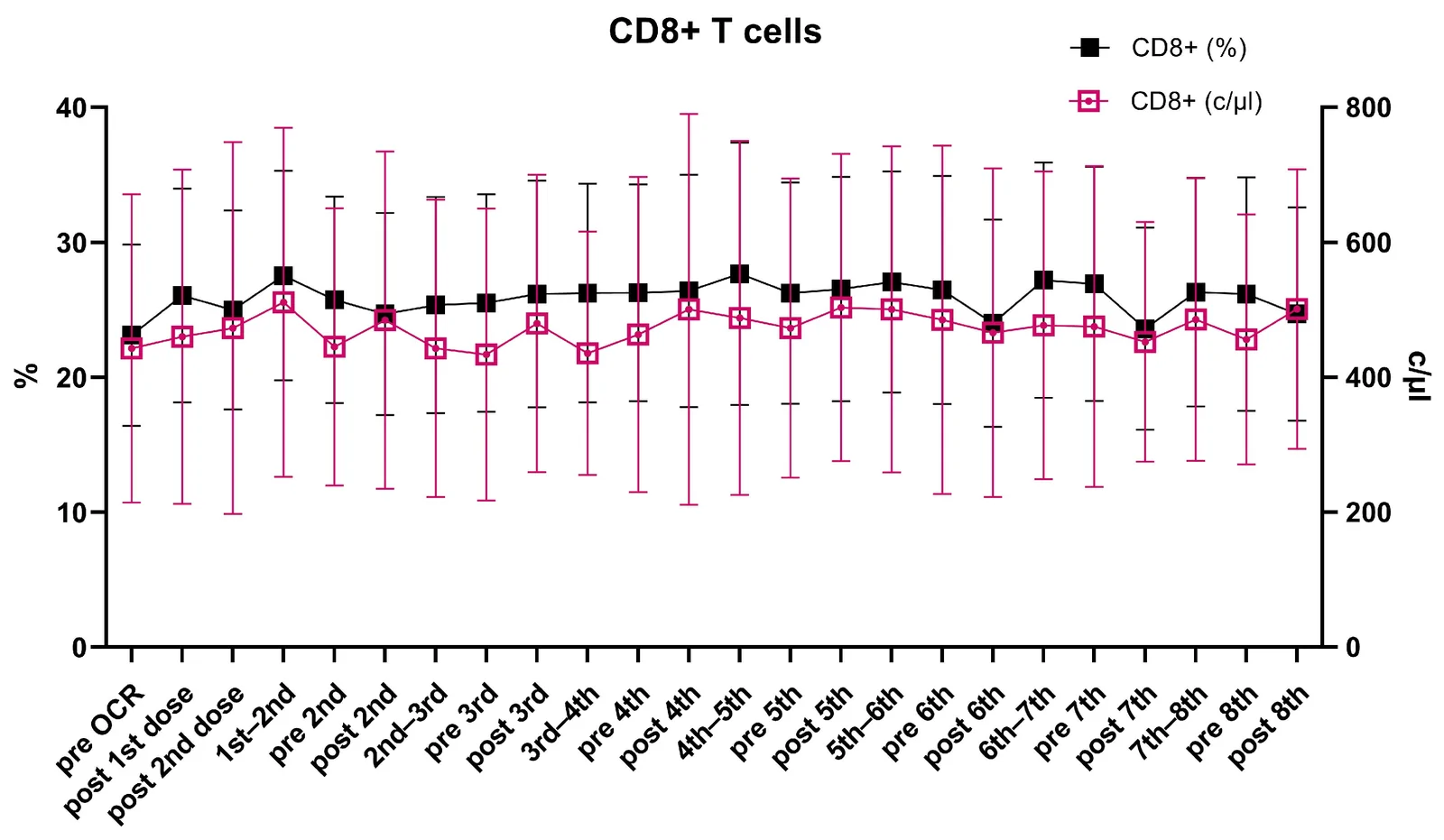
Longitudinal dynamics of CD8+ T cells during OCR treatment. Mean ± SD CD8+ T cell frequencies (% of total lymphocytes) and absolute cell counts (c/µL) measured at each study time-point throughout eight OCR cycles. Note that CD8+ T cell frequencies remained relatively stable throughout follow-up, although lower values were observed at post-6th, post-7th, and post-8th OCR infusions. Pre-OCR: baseline assessment before ocrelizumab initiation; post 1st dose and post 2nd dose: evaluations performed after the first and second doses of the initial infusion, respectively; time-points indicated by two consecutive infusion cycles (e.g., 2nd–3rd, 3rd–4th) represent intermediate inter-infusion assessments; thereafter, pre and post indicate evaluations performed 15 days before and 15 days after each subsequent OCR infusion, respectively.

**Figure 6:**
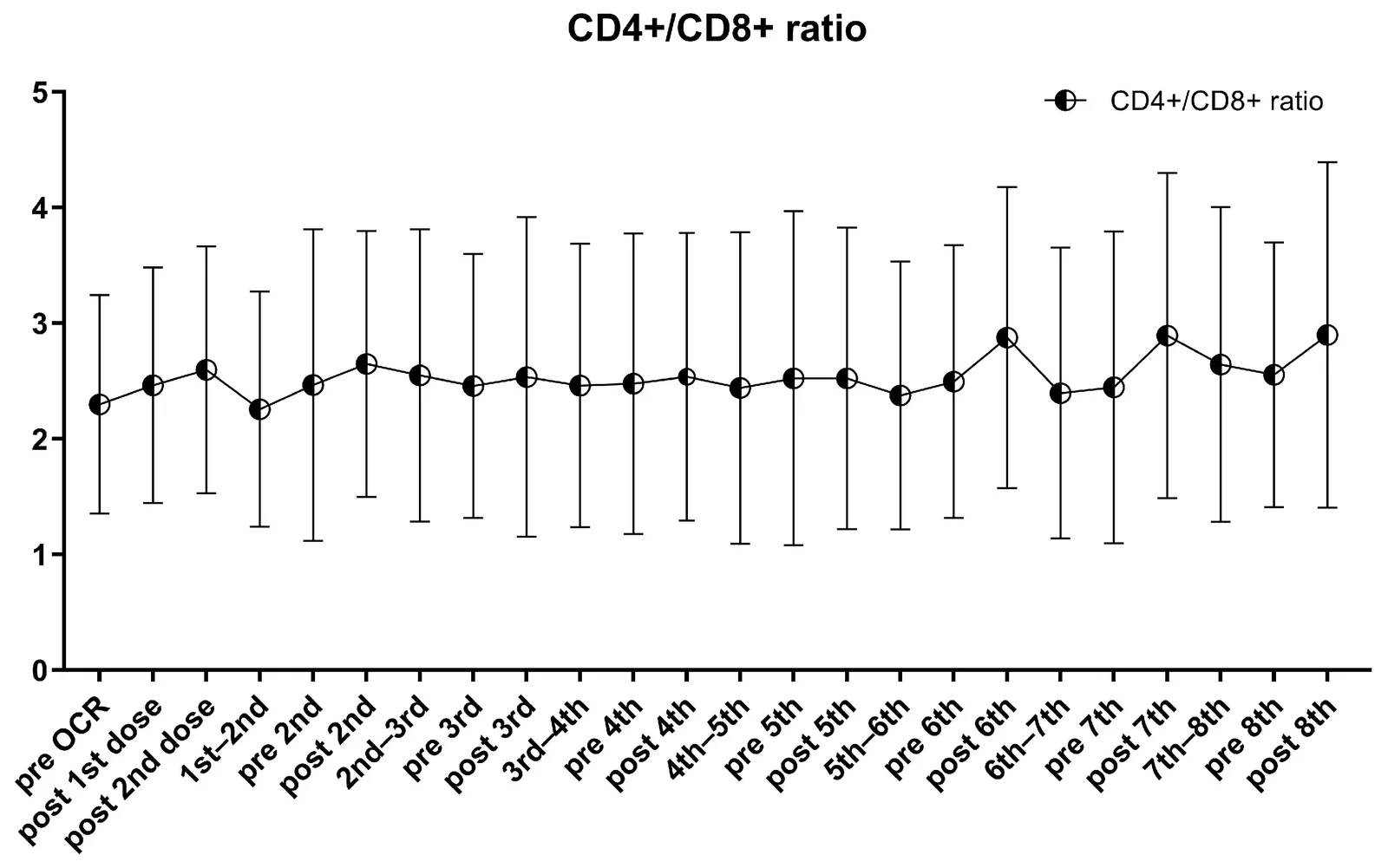
Longitudinal dynamics of the CD4+/CD8+ ratio during OCR treatment. Mean ± SD CD4+/CD8+ ratio measured at each study time-point throughout eight OCR infusion cycles. Note that the CD4+/CD8+ ratio increased during follow-up, reaching its highest values after the 6th OCR infusion, concomitantly with peak CD4+ T cell frequencies and lower CD8+ T cell frequencies. Pre-OCR: baseline assessment before ocrelizumab initiation; post 1st dose and post 2nd dose: evaluations performed after the first and second doses of the initial infusion, respectively; time-points indicated by two consecutive infusion cycles (e.g., 2nd–3rd, 3rd–4th) represent intermediate inter-infusion assessments; thereafter, pre and post indicate evaluations performed 15 days before and 15 days after each subsequent OCR infusion, respectively.

**Figure 7:**
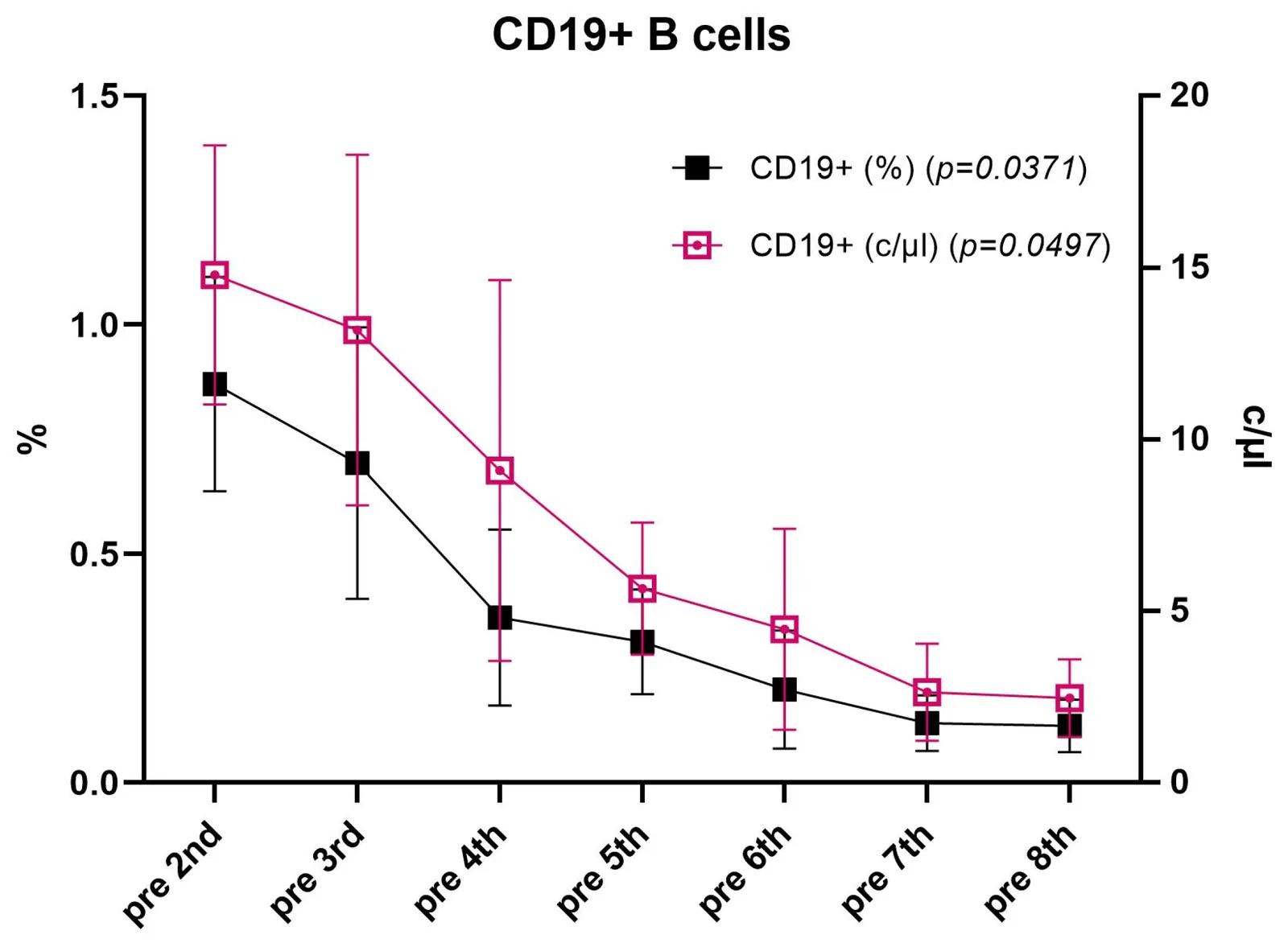
Longitudinal dynamics of pre-infusion CD19+ B cells. Mean ± SE CD19+ B cell frequencies (% of total lymphocytes) and absolute cell counts (c/µL) measured immediately before (pre) each OCR infusion throughout follow-up. Note that both parameters progressively declined over time, approaching near-zero values by the fourth year of treatment (overall longitudinal *p* = 0.0371 and *p* = 0.0497, respectively).

**Figure 8:**
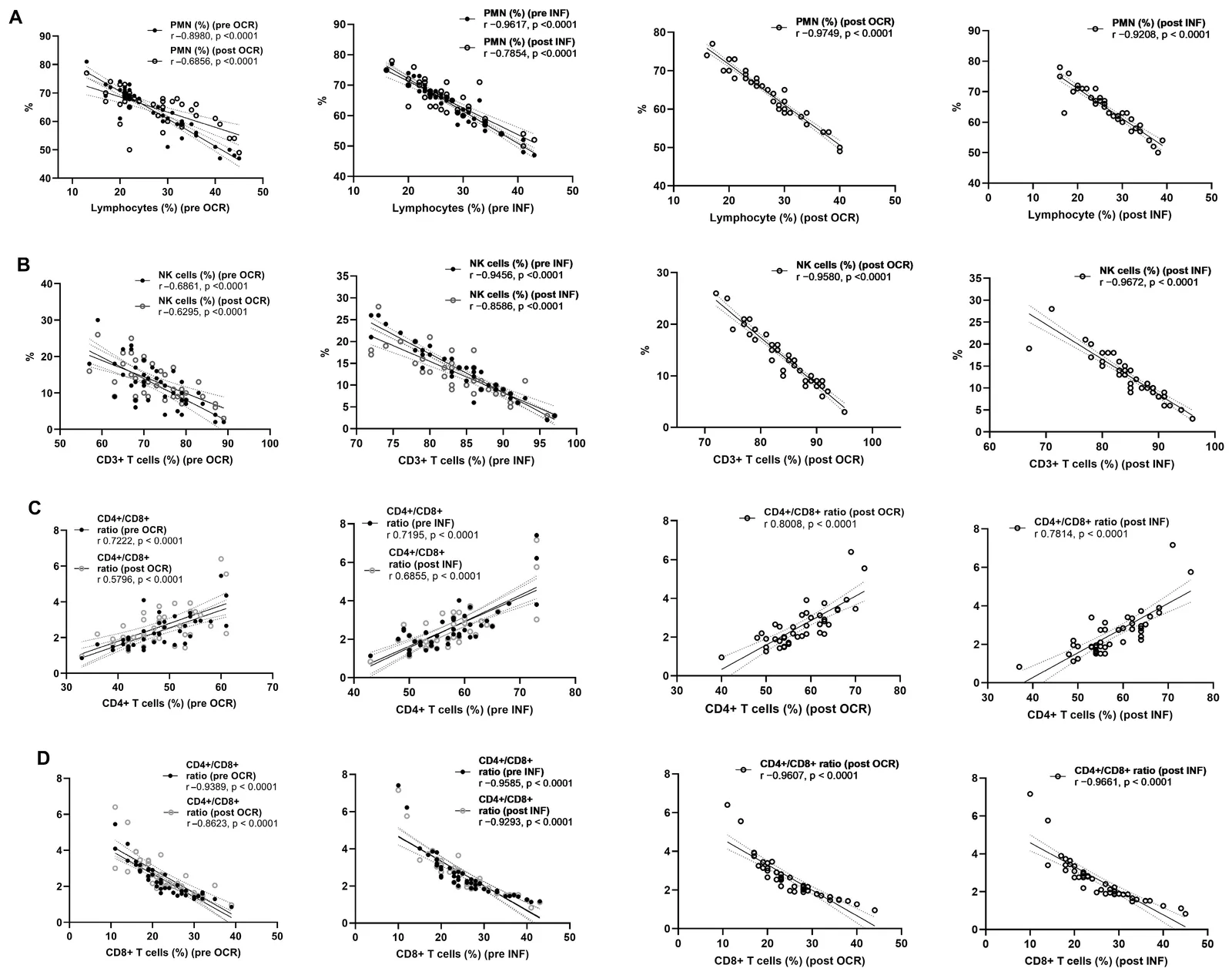
Inferential statistics with representative linear regression analyses of selected immune-cell correlations before and during OCR treatment. For each correlation, the first column shows pre-OCR correlated with both pre- and post-OCR values, the second column shows pre-INF correlated with both pre- and post-INF values, the third column shows post-OCR correlated with post-OCR values, and the fourth column shows post-INF correlated with post-INF values. (**A**) Inverse correlation between lymphocyte and PMN frequencies (pre-OCR vs. pre/post OCR: *r* = −0.8980 and *r* = −0.6856; pre-INF vs. pre/post INF: *r* = −0.9617 and *r* = −0.7854; post OCR vs. post OCR: *r* = −0.9749; post INF vs. post INF: *r* = −0.9208). (**B**) Inverse correlation between CD3+ T cell and NK cell frequencies (pre OCR vs. pre/post OCR: *r* = −0.6861 and *r* = −0.6295; pre INF vs. pre/post INF: *r* = −0.9456 and *r* = −0.8586; post OCR vs. post OCR: *r* = −0.9580; post INF vs. post INF: *r* = −0.9672). (**C**) Direct correlation between CD4+ T cell frequencies and the CD4+/CD8+ ratio (pre OCR vs. pre/post OCR: *r* = 0.7222 and *r* = 0.5796; pre INF vs. pre/post INF: *r* = 0.7195 and *r* = 0.6855; post OCR vs. post OCR: *r* = 0.8008; post INF vs. post INF: *r* = 0.7814). (**D**) Inverse correlation between CD8+ T cell frequencies and the CD4+/CD8+ ratio (pre OCR vs. pre/post OCR: *r* = −0.9389 and *r* = −0.8623; pre INF vs. pre/post INF: *r* = −0.9588 and *r* = −0.9293; post OCR vs. post OCR: *r* = −0.9607; post INF vs. post INF: *r* = −0.9661). Linear regression analyses showed *p*-values < 0.0001 for all correlations. Note that the major relationships among immune-cell subsets were consistently preserved before and after OCR initiation, as well as throughout successive infusion cycles, despite treatment-induced changes in their relative distribution.

**Table 1:** Descriptive and inferential statistics of leukocyte subpopulations before and after ocrelizumab treatment (pre-OCR vs. post-OCR) and ocrelizumab infusion (pre-INF vs. post-INF).

N = 51	Pre-OCR Mean ± SD (95% CI)	Post-OCR Mean ± SD (95% CI)	*p*-Value	Pre-INF Mean ± SD (95% CI)	Post-INF Mean ± SD (95% CI)	*p*-Value
WBC (c/μL)	6655 ± 2378 (5915–7416)	6978 ± 1830 (6400–7556)	*ns*	6624 ± 1987 (6027–7221)	7343 ± 2297 (6653–8033)	**<0.0001**
Lymphocytes (%)	27.37 ± 8.09 (24.81–29.92)	26.63 ± 5.84 (24.79–28.48)	*ns*	27.29 ± 6.02 (25.48–29.1)	25.96 ± 5.98 (24.16–27.75)	**0.0018**
Lymphocytes (c/μL)	1818 ± 682.5 (1602–2033)	1807 ±518.7 (1643–1971)	*ns*	1762 ± 507.3 (1610–1915)	1841 ± 581 (1667–2016)	**0.0091**
Monocytes (%)	6.93 ± 2.10 (6.26–7.59)	7.19 ± 1.28 (6.78–7.60)	*ns*	6.88 ± 1.3 (6.49–7.28)	7.26 ± 1.26 (6.88–7.64)	**0.0178**
Monocytes (c/μL)	452.3 ± 162.4 (401.1–503.6)	495.0 ± 134.6 (452.5–537.4)	**0.0168**	445.2 ± 130 (406.2–484.3)	516.4 ± 136 (475.6–557.3)	**<0.0001**
PMN (%)	63.44 ± 8.36 (60.8–66.08)	64.66 ± 6.31 (62.67–66.65)	*ns*	63.89 ± 6.59 (61.91–65.87)	65.13 ± 6.31 (63.24–67.03)	**0.0459**
PMN (c/μL)	4312 ± 1776 (3751–4872)	4606 ± 1457 (4147–5066)	*ns*	4333 ± 1534 (3872–4794)	4883 ± 1807 (4340–5426)	**<0.0001**
CD3+ T cells (%)	73.15 ± 7.46 (70.79–75.5)	84.88 ± 5.61 (83.11–86.65)	**<0.0001**	84.67 ± 6.11 (82.83–86.5)	85.11 ± 6.46 (83.17–87.05)	*ns*
CD3+ T cells (c/μL)	1329 ± 523.3 (1164–1494)	1536 ± 464.3 (1390–1683)	**0.0008**	1489 ± 468.1 (1348–1630)	1579 ± 532.6 (1419–1739)	**0.0009**
CD4+ T cells (%)	47.83 ± 6.65 (45.73–49.93)	57.80 ± 6.73 (55.68–59.93)	**<0.0001**	56.78 ± 7.17 (54.62–58.93)	57.58 ± 7.1 (55.44–59.71)	*ns*
CD4+ T cells (c/μL)	881.5 ± 392.7 (757.5–1005)	1053 ± 356 (941.1–1166)	**<0.0001**	1009 ± 349.5 (903.5–1114)	1073 ± 381.5 (958.6–1188)	**0.0002**
CD8+ T cells (%)	22.83 ± 6.51 (20.77–24.88)	25.49 ± 7.25 (23.2–27.78)	**<0.0001**	26.42 ± 7.92 (24.04–28.8)	25.93 ± 8.06 (23.51–28.35)	*ns*
CD8+ T cells (c/μL)	411.0 ± 183.3 (352.9–469.0)	455.7 ± 179.5 (399–512.4)	**0.0401**	457.9 ± 197.7 (398.5–517.3)	475.0 ± 231.0 (405.6–544.4)	*ns*
CD4+/CD8+ ratio	2.33 ± 0.94 (2.03–2.63)	2.57 ± 1.08 (2.23–2.91)	**0.0031**	2.50 ± 1.20 (2.13–2.86)	2.55 ± 1.16 (2.21–2.91)	*ns*
NK cells (%)	12.07 ± 6.12 (10.14–14.01)	12.98 ± 5.27 (11.31–16.64)	*ns*	13.04 ± 5.65 (11.34–14.74)	12.58 ± 5.77 (10.84–14.31)	*ns*
NK cells (c/μL)	220.3 ± 143.9 (174.9–265.7)	233.0 ± 117.2 (196.0–270.0)	*ns*	222.9 ± 119.9 (186.9–258.9)	228.5 ± 114.3 (194.1–262.8)	*ns*

Data are presented as mean ± SD (95% CI). Relative frequencies (% of WBCs) and absolute cell counts (cells/μL) are reported for each leukocyte subpopulation. Pre-OCR mean values refer to assessments performed before treatment initiation, whereas post-OCR mean values were calculated from all available on-treatment assessments up to the eighth OCR infusion cycle. Pre-INF and post-INF values correspond to mean measurements obtained 15 days before and 15 days after ocrelizumab infusion, respectively. Statistically significant *p*-values are shown in bold. OCR: ocrelizumab, WBC: white blood cells, PMN: polymorphonuclear leukocytes, NK: natural killer cells, c/μL: cells/microliters.

## Data Availability

The original contributions presented in this study are included in the article. Further inquiries can be directed to the corresponding author.

## Supplementary Materials

The following supporting information can be downloaded at: https://www.mdpi.com/article/10.3390/ph19101514/s1, Supplementary Data S1: Descriptive statistics and correlation matrices for all investigated leukocyte subsets and time-points, expressed as both cell frequencies (%) and absolute cell counts (c/µL); Supplementary Figure S1: Longitudinal dynamics of white blood cells during ocrelizumab treatment; Supplementary Figure S2: Longitudinal dynamics of lymphocytes during ocrelizumab treatment; Supplementary Figure S3: Longitudinal dynamics of granulocytes during ocrelizumab treatment; Supplementary Figure S4: Longitudinal dynamics of natural killer cells during ocrelizumab treatment; Supplementary Figure S5: Correlation matrices of pre- and post-ocrelizumab for all investigated leukocyte subsets expressed as both cell frequencies (%) and absolute cell counts (c/µL); Supplementary Figure S6: Correlation matrices of pre-, inter- and post-infusion for all investigated leukocyte subsets expressed as both cell frequencies (%) and absolute cell counts (c/µL).

## Abbreviations

The following abbreviations are used in this manuscript:

ADCC	Antibody-dependent cellular cytotoxicity
CDC	Complement-dependent cytotoxicity
CNS	Central Nervous System
CSF	Cerebrospinal fluid
DMT	Disease-Modifying Treatment
MS	Multiple Sclerosis
NKs	Natural Killer cells
OCR	Ocrelizumab
PMNs	Polymorphonuclear Leukocytes
PPMS	Primary-Progressive Multiple Sclerosis
RRMS	Relapsing-Remitting Multiple Sclerosis
WBC	White Blood Cells

## References

[1] Thompson A.J., Baranzini S.E., Geurts J., Hemmer B., Ciccarelli O. (2018). Multiple sclerosis. Lancet.

[2] Filippi M., Bar-Or A., Piehl F., Preziosa P., Solari A., Vukusic S., Rocca M.A. (2018). Multiple sclerosis. Nat. Rev. Dis. Primers.

[3] Hauser S.L., Bar-Or A., Comi G., Giovannoni G., Hartung H.P., Hemmer B., Lublin F., Montalban X., Rammohan K.W., Selmaj K. (2017). Ocrelizumab versus Interferon Beta-1a in Relapsing Multiple Sclerosis. N. Engl. J. Med..

[4] Montalban X., Hauser S.L., Kappos L., Arnold D.L., Bar-Or A., Comi G., de Seze J., Giovannoni G., Hartung H.P., Hemmer B. (2017). Ocrelizumab versus Placebo in Primary Progressive Multiple Sclerosis. N. Engl. J. Med..

[5] Havrdová E., Arnold D.L., Bar-Or A., Comi G., Hartung H.P., Kappos L., Lublin F., Selmaj K., Traboulsee A., Belachew S. (2018). No evidence of disease activity (NEDA) analysis by epochs in patients with relapsing multiple sclerosis treated with ocrelizumab vs interferon beta-1a. Mult. Scler. J. Exp. Transl. Clin..

[6] Vermersch P., Oreja-Guevara C., Siva A., Van Wijmeersch B., Wiendl H., Wuerfel J., Buffels R., Kadner K., Kuenzel T., Comi G. (2022). Efficacy and safety of ocrelizumab in patients with relapsing-remitting multiple sclerosis with suboptimal response to prior disease-modifying therapies: A primary analysis from the phase 3b CASTING single-arm, open-label trial. Eur. J. Neurol..

[7] Abbadessa G., Miele G., Maida E., Vanore E., Cipriano L., Coppola C., Lavorgna L., Bonavita S. (2023). Immunomodulatory effects of ocrelizumab and candidate biomarkers for monitoring treatment response in multiple sclerosis. Mult. Scler. J..

[8] Abbadessa G., Lepore M.T., Bruzzaniti S., Piemonte E., Miele G., Signoriello E., Perna F., De Falco C., Lus G., Matarese G. (2024). Ocrelizumab Alters Cytotoxic Lymphocyte Function While Reducing EBV-Specific CD8^+^ T-Cell Proliferation in Patients with Multiple Sclerosis. Neurol. Neuroimmunol. Neuroinflamm..

[9] Abbadessa G., Maida E., Miele G., Lavorgna L., Marfia G.A., Valentino P., De Martino A., Cavalla P., Bonavita S. (2022). Lymphopenia in Multiple Sclerosis patients treated with Ocrelizumab is associated with an effect on CD8 T cells. Mult. Scler. Relat. Disord..

[10] Garcia A., Dugast E., Shah S., Morille J., Lebrun-Frenay C., Thouvenot E., De Sèze J., Le Page E., Vukusic S., Maurousset A. (2023). Immune Profiling Reveals the T-Cell Effect of Ocrelizumab in Early Relapsing-Remitting Multiple Sclerosis. Neurol. Neuroimmunol. Neuroinflamm..

[11] Fernández-Velasco J.I., Kuhle J., Monreal E., Meca-Lallana V., Meca-Lallana J., Izquierdo G., Gascón-Giménez F., Sainz de la Maza S., Walo-Delgado P.E., Maceski A. (2021). Effect of Ocrelizumab in Blood Leukocytes of Patients with Primary Progressive MS. Neurol. Neuroimmunol. Neuroinflamm..

[12] Capasso N., Palladino R., Cerbone V., Spiezia A.L., Covelli B., Fiore A., Lanzillo R., Carotenuto A., Petracca M., Stanziola L. (2023). Ocrelizumab effect on humoral and cellular immunity in multiple sclerosis and its clinical correlates: A 3-year observational study. J. Neurol..

[13] Monteiro I., Nicolella V., Fiorenza M., Novarella F., Carotenuto A., Lanzillo R., Mauriello L., Scalia G., Castaldo G., Terracciano D. (2024). The ocrelizumab wearing-off phenomenon is associated with reduced immunomodulatory response and increased neuroaxonal damage in multiple sclerosis. J. Neurol..

[14] Vodă I.M., Tiu V.E., Răuță L., Ciucur P., Mușuroi A.I., Tomescu A.F., Humă N.L., Dobrițoiu F., Terecoasă E., Negreanu L. (2025). Ocrelizumab-induced colitis-critical review and case series from a Romanian cohort of MS patients. Front. Neurol..

[15] Emma C., Vesela P., Kay P., Huseyin H., Alison H., Mara S., Floriana A., Sara C. (2024). Psoriasiform dermatitis following ocrelizumab in relapsing-remitting multiple sclerosis: Case report and literature review. Mult. Scler. J..

[16] De Masi R., Orlando S. (2026). Ocrelizumab-Induced Brain Volume Dynamics in Relapsing-Remitting Multiple Sclerosis. Pharmaceuticals.

[17] Capasso N., Nozzolillo A., Scalia G., Lanzillo R., Carotenuto A., De Angelis M., Petruzzo M., Saccà F., Russo C.V., Brescia Morra V. (2021). Ocrelizumab depletes T-lymphocytes more than rituximab in multiple sclerosis. Mult. Scler. Relat. Disord..

[18] Willison A.G., Hagler R., Weise M., Elben S., Huntemann N., Masanneck L., Pfeuffer S., Lichtenberg S., Golombeck K.S., Preuth L.M. (2025). Effects of Anti-CD20 Antibody Therapy on Immune Cell Dynamics in Relapsing-Remitting Multiple Sclerosis. Cells.

[19] Friedli C., Krajnc N., Hammer H.N., Marti S., Zrzavy T., Evangelopoulos M.E., Kapsali I., Rommer P., Berger T., Chan A. (2024). Different lymphocyte counts of multiple sclerosis patients treated with ofatumumab and ocrelizumab: A retrospective observational study. J. Cent. Nerv. Syst. Dis..

[20] Zanghì A., Di Filippo P.S., Papale M., Rutigliano C., Moretti M.C., Avolio C., Corso G., D’Amico E. (2025). Modeling lymphocyte subset dynamics after ublituximab therapy in patients with multiple sclerosis: An Italian prospective study. Front. Immunol..

[21] Fox E., Lovett-Racke A.E., Gormley M., Liu Y., Petracca M., Cocozza S., Shubin R., Wray S., Weiss M.S., Bosco J.A. (2021). A phase 2 multicenter study of ublituximab, a novel glycoengineered anti-CD20 monoclonal antibody, in patients with relapsing forms of multiple sclerosis. Mult. Scler. J..

[22] Martin S.J., Guenette M., Oh J. (2024). Evaluating the Therapeutic Potential of Ublituximab in the Treatment of MS: Design, Development and Place in Therapy. Drug Des. Devel. Ther..

[23] De Masi R., Orlando S. (2020). IFI35 as a biomolecular marker of neuroinflammation and treatment response in multiple sclerosis. Life Sci..

[24] De Masi R., Orlando S. (2021). GANAB as a Novel Biomarker in Multiple Sclerosis: Correlation with Neuroinflammation and IFI35. Pharmaceuticals.

[25] De Masi R., Orlando S., Bagordo F., Grassi T. (2021). IFP35 Is a Relevant Factor in Innate Immunity, Multiple Sclerosis, and Other Chronic Inflammatory Diseases: A Review. Biology.

[26] De Masi R., Orlando S. (2022). GANAB and N-Glycans Substrates Are Relevant in Human Physiology, Polycystic Pathology and Multiple Sclerosis: A Review. Int. J. Mol. Sci..

[27] Fischer S., Proschmann U., Akgün K., Ziemssen T. (2021). Lymphocyte Counts and Multiple Sclerosis Therapeutics: Between Mechanisms of Action and Treatment-Limiting Side Effects. Cells.

[28] Maeda N., Sekigawa I., Iida N., Matsumoto M., Hashimoto H., Hirose S. (1999). Relationship between CD4^+^/CD8^+^ T cell ratio and T cell activation in systemic lupus erythematosus. Scand. J. Rheumatol..

[29] Sweiss N.J., Salloum R., Gandhi S., Alegre M.L., Sawaqed R., Badaracco M., Pursell K., Pitrak D., Baughman R.P., Moller D.R. (2010). Significant CD4, CD8, and CD19 lymphopenia in peripheral blood of sarcoidosis patients correlates with severe disease manifestations. PLoS ONE.

[30] Lu W., Mehraj V., Vyboh K., Cao W., Li T., Routy J.P. (2015). CD4:CD8 ratio as a frontier marker for clinical outcome, immune dysfunction and viral reservoir size in virologically suppressed HIV-positive patients. J. Int. AIDS Soc..

[31] Thompson A.J., Banwell B.L., Barkhof F., Carroll W.M., Coetzee T., Comi G., Correale J., Fazekas F., Filippi M., Freedman M.S. (2018). Diagnosis of multiple sclerosis: 2017 revisions of the McDonald criteria. Lancet Neurol..

